# Placement Work Experience May Mitigate Lower Achievement Levels of Black and Asian vs. White Students at University

**DOI:** 10.3389/fpsyg.2017.01518

**Published:** 2017-09-12

**Authors:** Elisabeth Moores, Gurkiran K. Birdi, Helen E. Higson

**Affiliations:** ^1^School of Life and Health, Aston University Birmingham, United Kingdom; ^2^Aston Business School, Aston University Birmingham, United Kingdom

**Keywords:** attainment gap, placement, internship, University, ethnicity, performance, achievement, BME

## Abstract

Ethnic minority groups have been shown to obtain poorer final year degree outcomes than their majority group counterparts in countries including the United States, the United Kingdom and The Netherlands. Obtaining a lower degree classification may limit future employment prospects of graduates as well as opportunities for higher level study. To further investigate this achievement gap, we analyzed performance levels across three academic years of study of 3,051 Black, Asian and White students from a United Kingdom University. Analyses of covariance investigated effects of ethnicity and work placement experience (internships) on first, second and final year marks, whilst statistically controlling for a number of factors thought to influence achievement, including prior academic performance. Results demonstrated superior achievement of White students consistently across all years of study. Placement experience reduced, but did not eliminate, the size of the achievement gap exhibited by final year students. Sex, parental education and socioeconomic status had no significant main effects. Female students showed a more complex pattern of results than males, with Black females not showing the same final year uplift in marks as their Asian and White counterparts. Implications and possible explanations are discussed.

## Introduction

Ethnic minority groups have been reported to have final year degree outcomes that are inferior to their majority group counterparts in The Netherlands (Van Den Berg and Hofman, 2005; Severiens and Wolff, 2008), the United States (e.g., Betts and Morell, 1999) and the United Kingdom (HEFCE, 2015). Whilst overall proportions of University students receiving ‘good’ (first or upper second classification) degrees in the United Kingdom have increased over the past decade, the gap between the proportions of White British students achieving at this level in comparison with United Kingdom-domiciled students from Black and Minority Ethnic (BME) groups remains (75.6% vs. 60.4%: Equality Challenge Unit, 2015). This is particularly disturbing in the context that the implications of obtaining a lower degree classification are potentially enduring. An ever increasing number of graduate employers require applicants to hold at least a upper second (2.1) classified degree (77%: Association Graduate Recruiters, 2015), and at many institutions holding a degree with a lower second (2.2) classification can also prevent graduates from undertaking higher level University study. Addressing the attainment gap at an institutional level is therefore an ethical imperative.

Prior attainment, although a key factor in predicting degree outcomes, does not fully account for the differences between ethnic groups (Leslie, 2005; Broecke and Nicholls, 2007; Richardson, 2008, 2015; Fletcher and Tienda, 2010; HEFCE, 2015). Broecke and Nicholls (2007) conducted a large scale study which investigated 65,000 United Kingdom qualifiers and found that BME students obtained lower degree results than their white counterparts, even when controlling for prior attainment, age, gender, and discipline. In the same study, students obtaining entry to university via ‘academic’ (e.g., A-level, International Baccalaureate) rather than ‘vocational’ (e.g., Business and Technology Council: BTEC) qualifications tended to achieve higher marks. However, Broecke and Nicholls (2007) did not control for parental education experience, a factor which Connor et al. (2004) suggested significantly differs between ethnic groups. Similarly, in the United States, Fletcher and Tienda (2010) found that controlling for prior attainment reduced, but did not eliminate, gaps between White students and their Hispanic and Black counterparts. They instead considered high school ‘quality’ as an explanatory factor. Previous research has investigated various potential causes of the gap in attainment between BME and White students, often controlling for prior performance. However, whilst a number of factors contributing to the poorer attainment of BME students at University level have been identified, none have been able to fully account for the observed gaps between White and BME students.

In England, ethnic minority groups are now – on average – more likely to go to university than their white counterparts (Crawford and Greaves, 2015). However, in the majority of institutions, non-white students remain a minority. Sadly, those institutions with higher proportions of BME students appear to have *greater* differentials in attainment, with the exception of Russell Group^1^ Universities where a benefit of increased representation is observed (Fielding et al., 2008). Many of the universities that BME students go to are modern institutions; the Russell Group Universities have over 12% more white students than the Million+^2^ group of Universities (Equality Challenge Unit, 2015). Controversially, Boliver (2016) argues that admissions policies at some Russell Group Universities may even be biased against ethnic minority applicants, further compounding the situation. Although the United Kingdom based University and Colleges Admissions Service (UCAS), which handles and analyses almost all admissions to United Kingdom Universities, dispute this interpretation they have recently started to publish such equality data for each individual University to consider. Most previous research on the BME attainment gap has been conducted in institutions where BME students are a minority, or used large datasets which have combined data from a number of institutions with very different characteristics. Whilst qualitative research (see, e.g., Read et al., 2003) can help to elucidate the experiences of BME students in this context, it is impossible to quantify to what extent these experiences as ‘a minority’ actually impact on academic achievement. In a synthesis of the literature, Singh (2009, p. 29) suggests that *“a recurring theme in many studies is the lack of support and isolation that many BME students feel.”*

The majority of the studies looking at the BME attainment differences in Higher Education either focus on the attainment gap for qualifying students, or upon student retention and attrition rates in earlier years of study (e.g., Connor et al., 2004; Broecke and Nicholls, 2007; Fielding et al., 2008; Richardson, 2008; Meeuwisse et al., 2010b). Little research has investigated whether the gap occurs earlier on in academic study (i.e., post-entry but pre-graduation) or more specifically whether the gap changes throughout the period of study. Previous research (e.g., Thiele et al., 2016) has suggested that many entry level differences may be narrowed *by* the final year of study, but little is known about how these effects influence performance across the different study years whilst *at* University. Critically, this information may provide clues to help our understanding of the causes of the gaps, as well as how best to reduce them. Previous research has investigated a variety of possible differences between different ethnic groups in conceptions of learning (Richardson, 2010), entry qualifications (e.g., Richardson, 2008), intentions to persist (Eimers and Pike, 1997), and sense of belonging, integration and prejudice (Nora and Cabrera, 1996; Read et al., 2003; Severiens and Wolff, 2008; Meeuwisse et al., 2010a). To date, no single factor has been able to fully account for the gap.

Several researchers have reported that work experience undertaken whilst on a placement year or internship during students’ degree programmes has a positive effect on final year marks when they return to university (e.g., Gomez et al., 2004; Mandilaras, 2004; Rawlings et al., 2005; Reddy and Moores, 2006; Mendez, 2008; Surridge, 2009; Green, 2011; Mansfield, 2011; Crawford and Wang, 2016), although see also Duignan (2002). Jones et al. (2015) reported on the beneficial effects of a work placement on final year performance across two United Kingdom Universities, despite accounting for the self-selection effects of opting to complete a placement. Reddy and Moores (2012) showed the benefit held at Aston University regardless of ethnicity, sex and socioeconomic background, but also noted that these factors influence whether or not students actually choose to take an optional placement year. Blasko et al. (2002) suggest that work experience during a degree programme has a larger positive effect on employment for lower socio-economic status groups – the work experience helps to bridge the divide that was already present. Moores and Reddy (2012) also showed the career benefit of placement experience for psychology students. Despite the clear impact of a placement year on final year attainment and employment success, and the known differential uptake of this experience across ethnicities, placement experience has not been previously considered as a potential moderating variable for the BME achievement gap.

Thus, the present study explored data from a single institution, in which White students are a minority, to examine whether a BME attainment gap still occurs in a highly ethnically diverse student environment, how any BME attainment gap is manifested over the different years of academic study and whether placement experience narrows the gap. In addition, we split students into higher and lower entry tariff groups in order to investigate whether students with higher vs. lower prior academic achievement were affected differentially by any gap. A number of other variables known to influence attainment were also included in the analyses in order to examine the any potential interplay between factors and to provide statistical control for differences in our sample unrelated to students’ ethnic backgrounds. Richardson (2008), for example, reported a more pronounced BME attainment gap in women than in men and Thiele et al. (2016) reported some persisting effects of socio-economic differences on achievement. End of year (stage average) marks were used to ascertain the size of the gap in each year of study. Our hypotheses were that: (i) white and BME students would have different levels of achievement, despite statistically controlling for prior attainment and other factors known to influence achievement, (ii) the size of the BME achievement gap would increase across the years of study, (iii) placement experience would reduce the size of the BME achievement gap and (iv) the BME achievement gap would be larger amongst students with lower prior attainment. In addition, we expected to see better performance of females (vs. males) and a reduction in the influence of prior attainment across the years of study.

## Materials and Methods

### Sample Data

Aston University is an ethnically diverse institution with a high population of Asian students in comparison to other United Kingdom Higher Education Institutions (35% vs. around 8% nationally) and – unusually – an overall white minority (36% vs. around 80% nationally: Equality Challenge Unit, 2015). Aston University is not affiliated to the Russell Group, or the Million+ group. As a former technical college originally created by the employers of Birmingham in 1895, it gained its University status in 1966. Aston University prides itself on its placement year provision and consequential high rates of graduate employability, with many of its students taking a placement year as part of their degree. Undergraduate student performance (end of year or ‘stage average mark’) and demographic data were obtained via Aston University’s electronic records system for graduates from academic years 2010–11 to 2014–15. The initial sample comprised 5,740 records with information on: degree classification, first year average mark, second year average mark, final year average mark, sex, ethnicity, award year, whether or not the student took a placement, socio-economic status, parental educational background, UCAS entry tariff, type of school attended prior to university and home or overseas fee status.

In order to match students from various backgrounds as closely as possible the following exclusions were made: (i) Students with overseas tuition fee status: this group might be expected to have a different language and acclimatization background from Home students; (ii) Students from independent schools: this group (<10%) shows quite a different pattern in terms of ethnicity and performance and previous analyses have suggested that these students do not typically perform as well as other students with similar entry qualifications (e.g., HEFCE, 2003; Thiele et al., 2016); (iii) Students entering with qualifications other than A-levels: students with BTEC qualifications in particular tend to underperform relative to their peers with similar UCAS tariffs (e.g., Broecke and Nicholls, 2007); (iv) Students with missing or refused data on parental educational background: we wanted to include this as a dichotomous (yes/ no) variable for simplicity so omitted those without data; and (v) Students who reported being from ‘mixed’ or ‘other’ backgrounds, or refusing information: these groups were relatively small in number in our sample so were omitted in order to provide a more reliable analysis. The included sample (*n* = 3,051) had the following characteristics: 56% female, 43% White/50% Asian/7% Black, 46% had taken a placement and 44% were first generation at University.

### Measures

Stage average mark was the dependent variable in all analyses and was expressed as a percentage with 100% being the maximum mark achievable. Although Universities often use a variety of methods to determine a student’s degree classification, students can be assured of a first class degree with a mark of 70% and above, an upper second class degree with a mark of 60% and above and a lower second class degree with a mark of 50% and above. Students with between 40 and 50% are awarded a third class degree and below 40% a degree is not normally awarded.

Sex was coded as male or female. Ethnicity data were recorded as declared by the students themselves using the 18 categories used for United Kingdom census data, but later grouped into the superordinate categories of “Asian or Asian British,” “Black/African/Caribbean/Black British,” “White,” “Mixed/Multiple ethnic groups” and “Other.” Whether or not a student had taken a placement was coded as “yes” or “no”. Socioeconomic status contained (arguably) ordinal data based on occupation and was coded from one to eight based on the National Statistics Socio-economic classification (NS-SEC) analytic classes (1 = Higher managerial, administrative and professional occupations and 8 = Never worked and long-term unemployed).

UCAS entry tariffs ranged from 40 to 480 with a mean of 309. UCAS tariffs are scores given to a variety of qualifications based on the ‘size of’ (effort required) and the ‘achievement in’ (performance level) those qualifications. UCAS entry tariffs were used as a measure of prior academic achievement. The calculation of these tariffs has recently changed, but for the data included in our analyses, an A level with a grade A would have been awarded a UCAS tariff of 120 points, an A level with a grade B 100 points and a grade C 80 points. As levels attracted half the number of points as their A level equivalents. In addition to the total UCAS tariff scores used in the analysis as a continuous covariate, we also created a ‘UCAS excellence’ variable to split students into UCAS higher (320 points or above: roughly corresponding to ABB A levels or above) vs. lower (lower than 320 points) performing students. In 2014, the United Kingdom government requested that restrictions usually applied to student recruitment to Universities be lifted for students with ‘very high’ grades prior to entry – this included students with ABB A level grades and above. Fifty-two percentage of our sample were defined as ‘high UCAS excellence.’

### Analyses

The data were coded and statistically analyzed using IBM SPSS version 23. Once coded, ANCOVAs were used to analyze the data. Stage average mark was the dependent variable. The main independent variables of interest were sex, ethnicity, placement status, parental education, year of study and UCAS excellence. Socioeconomic status and UCAS entry tariff were used as covariates in the ANCOVA as a statistical control for their influence. The first ANCOVA analyzed only final year student data. The second ANCOVA used year of study (First, Second or Final) as an additional independent variable.

## Results

### Exploration of the Final Year Attainment Gap

**FIGURE 1:**
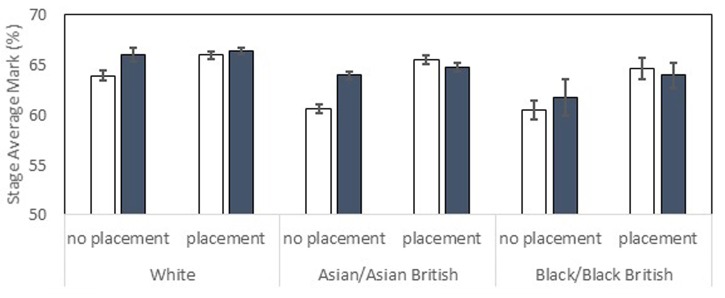
Stage average mark percentage for the final year shown by ethnicity, placement status and UCAS excellence. High UCAS excellence students are shown with shaded bars. Low UCAS excellence students are shown with unshaded bars. Standard error bars shown.

**Figure 1** shows the final stage average marks split by *ethnicity, placement* and *UCAS excellence*. White students and those that did placements achieved higher marks. White students (*M* = 65.26, *SE* = 0.24) achieved higher marks than both Asian (*M* = 63.7, *SE* = 0.21) and Black (*M* = 62.81, *SE* = 0.65) students. Students who had taken a placement (*M* = 64.24, *SE* = 0.31) performed better than those who had not (*M* = 63.82, *SE* = 0.32). The BME achievement gap was smaller amongst students who had taken a placement. Variables analyzed in a between subjects ANCOVA investigating effects on final stage average marks were: *sex* (male/female), *ethnicity* (White/Asian/Black), previous *parental education* in HE (yes/no), *UCAS excellence* (high/low), *UCAS entry tariff* (covariate), *socioeconomic status* (covariate) and whether or not the student had taken a *placement* (yes/no).

#### Main Effects

Significant main effects of *ethnicity* [*F*(2,2999) = 21.51, *p* < 0.001, $\eta_{p}^{2}$ = 0.014] and *placement* [*F*(1,2999) = 26.97, *p* < 0.001, $\eta_{p}^{2}$ = 0.009] were found as described above. *UCAS excellence* did not have a significant main effect [*F*(2,2999) = 2.84], but *UCAS entry tariff* was a significant covariate [*F*(1,2999) = 54.89, *p* < 0.001, $\eta_{p}^{2}$ = 0.018]; there was a positive correlation between UCAS entry tariff and achievement. *Socioeconomic status* was not a significant covariate and neither *parental education* nor *sex* had significant main effects (*F*s < 1*)*.

#### Interaction Effects

The *placement* × *ethnicity* interaction was significant [*F*(2,2999) = 3.48, *p* < 0.05, $\eta_{p}^{2}$ = 0.002]; the BME achievement gap was reduced amongst students who had taken a placement. *Placement*× *UCAS excellence* was also significant [*F*(1,2999) = 6.84, *p* < 0.01, $\eta_{p}^{2}$ = 0.002]; the positive effect of a placement on achievement was larger in students with low UCAS excellence. The interactions between *sex* × *ethnicity* [*F*(2,2999 = 2.85, *p* = 0.058, $\eta_{p}^{2}$ = 0.002] and *placement*× *sex*× *UCAS excellence*× *parental education* [*F*(1,2999 = 2.57, *p* = 0.052, $\eta_{p}^{2}$ = 0.001] narrowly missed significance. All other effects were not significant.

In summary, the widely reported BME achievement gap was replicated in this sample, with White students achieving higher marks than both Black and Asian students. Although effects were relatively small, it is noteworthy that whereas UCAS entry tariff explained 1.8% of the variance in the data, ethnicity explained 1.4%. However, the BME achievement gap was smaller in students who had taken a placement, with Black and Asian students benefitting from this experience more than White students. Placement experience was associated with higher final stage average marks, in particular amongst students who had entered University with lower UCAS excellence.

### Exploration of the Attainment Gap across the Years of Study

**FIGURE 2:**
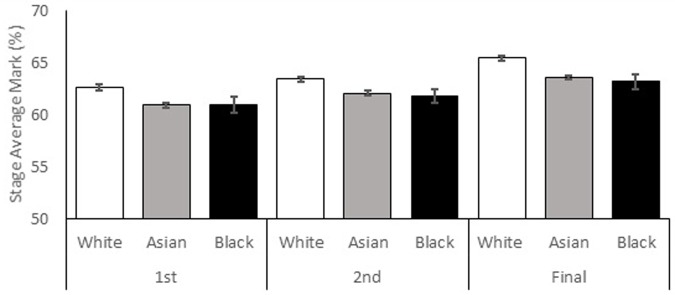
Stage average mark percentage shown by ethnicity and year of study. Standard error bars shown.

**FIGURE 3:**
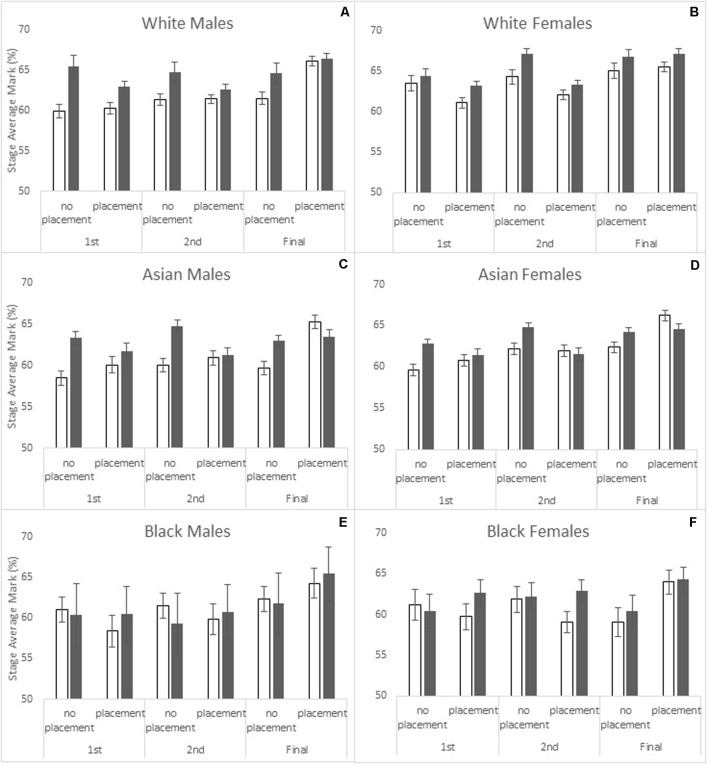
Stage average mark percentage shown by placement, UCAS excellence, and year of study for the different ethnicity and gender groups **(A–F)**. High UCAS excellence bars are shaded, low UCAS excellence unshaded. Standard error bars shown.

**Figure 2** shows the mean stage average marks by *study year* and *ethnicity*. A general increase in performance over the years of study can be seen for all ethnic groups investigated, as well as higher overall achievement by White students. White students (*M* = 63.82, *SE* = 0.23) performed better than Asian (*M* = 62.20, *SE* = 0.19) and Black (*M* = 62.13, *SE* = 0.62) students. Final year performance (*M* = 64.06, *SE* = 0.26) was higher than second year (*M* = 62.43, *SE* = 0.26) performance and second year was higher than first year (*M* = 61.66, *SE* = 0.28) performance. **Figures 3A–F** shows how students who have taken a placement improve their marks more in the final year than those who have not. In addition, the difference between high and low UCAS excellence students is markedly reduced (and sometimes reversed) in final year students who have taken a placement. The overall increase in performance over the years of study is not experienced equally by sexes and ethnic groups.

In order to explore the BME achievement gap by academic *study year*, first, second and final year performance were examined using a mixed measures ANCOVA. As before, other variables included in the analysis were *sex, ethnicity, parental education, UCAS excellence, UCAS entry tariff* (covariate), *socioeconomic status* (covariate) and *placement*.

#### Main Effects

Significant main effects of *study year* [*F*(2,5386) = 5.85, *p* < 0.01, $\eta_{p}^{2}$ = 0.002], *UCAS entry tariff* [*F*(1,2693) = 82.62, *p* < 0.001, $\eta_{p}^{2}$ = 0.030], *ethnicity* [*F*(2,2693 = 15.00, *p* < 0.001, $\eta_{p}^{2}$ = 0.011], and *UCAS excellence* [*F*(1,2693) = 7.67, *p* < 0.01, $\eta_{p}^{2}$ = 0.003] were found. Main effects of *socioeconomic status, placement, sex* and *parental education* were not significant (*F*s < 1).

#### Interaction Effects

Crucially for our hypotheses, there was no significant *study year*× *ethnicity* interaction [*F*(4,5386) = 1.45]; the BME achievement gap was not increasing by study year, but neither was it decreasing. The *study year* × *placement* interaction was significant [*F*(2,5386) = 44.59, *p* < 0.001, $\eta_{p}^{2}$ = 0.016]; there was a steep increase in performance from the second academic year to the final year in those who undertook a placement between these two academic years, and a decrease in performance for the same period for those who did not take a placement. *Study year* × *UCAS* excellence was also significant [*F*(2,5386) = 3.05, *p* < 0.05, $\eta_{p}^{2}$ = 0.001]; there was a bigger difference between high and low UCAS excellence in the first academic year compared to other years, suggesting a reduction of influence of prior performance over time. There was a significant *ethnicity* × *placement* × *UCAS excellence* interaction [*F*(2,2693) = 3.61, *p* < 0.05, $\eta_{p}^{2}$ = 0.003]. This mirrored the pattern already reported above; placement experience was associated with better performance overall for BME students and lower UCAS excellence students. *Study year* × *sex* × *UCAS excellence* narrowly missed significance [*F*(4,5386) = 2.80, *p* = 0.061, $\eta_{p}^{2}$ = 0.001], but s*tudy year* × *sex* × *UCAS excellence* × *parental education* [*F*(2,5386) = 3.54, *p* < 0.05, $\eta_{p}^{2}$ = 0.001] and *study year* × *sex* × *parental education* × *ethnicity*× *placement* [*F*(4,5386) = 2.62, *p* < 0.05, $\eta_{p}^{2}$ = 0.002] interactions were significant. These interactions are explored further below. Other interactions were not significant.

In order to understand better the four and five way interactions reported above, further mixed measures ANCOVA analyses were conducted on male and female students separately. For male students (**Figures 3A,C,E**) there were significant effects of *ethnicity* [*F*(2,1185) = 4.58, *p* < 0.05, $\eta_{p}^{2}$ = 0.008], *UCAS entry tariff* [*F*(1,1185) = 29.34, *p* < 0.001, $\eta_{p}^{2}$ = 0.024] and *UCAS excellence* [*F*(1,1185) = 4.23, *p* < 0.05, $\eta_{p}^{2}$ = 0.004]. The main effects of *placement* [*F* < 1] and *study year* [*F*(2,2370) = 2.26] were not significant, but there was a significant *placement* × *study year* interaction [*F*(2,1185) = 14.03, *p* < 0.001, $\eta_{p}^{2}$ = 0.012]. Male students who did not take a placement prior to their final year showed little improvement in marks in their final year, whereas those who had done placements showed an average mark improvement of over 3%. There was also a *study year* × *UCAS excellence* interaction [*F*(2,1185) = 3.29, *p* < 0.05, $\eta_{p}^{2}$ = 0.003]. As already described above, the gap between high and low UCAS excellence male students was largest in year 1 and smallest in the final year, although high UCAS excellence students consistently achieved the highest marks. Other main effects and interactions were not significant.

For female students (**Figures 3B,D,F**) there were significant effects of *ethnicity* [*F*(2,1506) = 13.70, *p* < 0.001, $\eta_{p}^{2}$ = 0.018] and *UCAS entry tariff* [*F*(1,1506) = 56.38, *p* < 0.001, $\eta_{p}^{2}$ = 0.036], but *UCAS excellence* narrowly missed significance [*F*(1,1506) = 3.58, *p* = 0.061]. In contrast to the males, females had a significant main effect of *study year* [*F*(2,3012) = 3.66, *p* < 0.05, $\eta_{p}^{2}$ = 0.002], showing consistent improvement from first to final year of study, and of *parental education* [*F*(1,1506) = 3.99, *p* < 0.05, $\eta_{p}^{2}$ = 0.003], with lower performance amongst first generation female students. As with the males, the main effects of *placement* [*F* < 1] and *socioeconomic status* [*F*(1,1506) = 1.15] were not significant.

In terms of interaction effects for the female students, there was a significant *study year* × *placement* interaction [*F*(2,3012) = 45.73, *p* < 0.001, $\eta_{p}^{2}$ = 0.029] which showed that females who had taken placements performed at a slightly lower level than those who had not in both the first and second years of study, but higher in the final year (following the placement). In contrast to the males, the *study year* × *UCAS excellence* interaction was not significant [*F* < 1]. However, the *study year* × *ethnicity* interaction was significant [*F*(4,1506) = 3.80, *p* < 0.01, $\eta_{p}^{2}$ = 0.005] and showed that Black females were not experiencing the uplift in marks in the final year experienced by both White and Asian females. The *ethnicity* × *placement* interaction narrowly missed significance [*F*(2,1506) = 2.90, *p* = 0.055, $\eta_{p}^{2}$ = 0.004] but the *ethnicity* × *placement* × *UCAS excellence* interaction was significant [*F*(2,1506) = 3.19, *p* < 0.05, $\eta_{p}^{2}$ = 0.004]. This suggested that – across all study years – White high UCAS excellence females achieved higher marks then low UCAS excellence females, regardless of placement status. For Asian females, the benefit of being in the high UCAS excellence group was only exhibited amongst students who did not do placements, whereas for Black females being in the high UCAS excellence group was a benefit only amongst those who did do placements. A *study year* × *ethnicity* × *UCAS excellence* interaction [*F*(4,1506) = 2.52, *p* < 0.05, $\eta_{p}^{2}$ = 0.003] showed that Asian females with low UCAS excellence caught up with their high UCAS excellence counterparts by the final year, whereas in White females the gap remained constant (in Black females the size of the gap was not significant). A *study year*× *UCAS excellence* × *placement* interaction [*F*(2,1506) = 3.43, *p* < 0.05, $\eta_{p}^{2}$ = 0.002] showed that in female students who had done placements, high and low UCAS excellence students performed at a similar level by the final year, whereas in those who had not done placements a differential in performance was still present. A *study year*× *ethnicity*× *placement*× *parental education* interaction [*F*(4,1506) = 3.67, *p* < 0.01, $\eta_{p}^{2}$ = 0.005] showed that the final year decline in performance in Black females was principally associated with those who had not done a placement and whose parents had not had a university level education. Other effects and interactions were not significant.

In summary, the situation for females was far more complex than for that of males, with multiple factors – including parental education, placement experience, UCAS excellence and ethnicity – influencing student attainment.

## Discussion

Consistent with our first hypothesis, even at a highly multi-cultural university where white students are a minority – and with a number of critical variables statistically controlled – White students still out-performed their Asian and Black counterparts in terms of final year marks. This worrying result replicates findings reported across the sector and reflects previous literature (e.g., Broecke and Nicholls, 2007; Fielding et al., 2008; Richardson, 2008) on the BME attainment gap in Higher Education. Arguably, some reassurance could come from the finding that, contrary to our second hypothesis, the BME achievement gap did not increase by year of study – at least not overall – suggesting that the university experience was not exacerbating the gap over time. However, nor was the gap decreasing, despite a general trend toward higher marks across the student population in the final year and evidence that the influence of other critical factors such as prior performance did decrease in some groups over the years of study (see also Thiele et al., 2016). Moreover, it could be considered of even greater concern that the BME achievement gap is already present in the first year of University study, despite the employment of statistical controls for entry qualifications. Furthermore, for Black females, the gap did grow, as this group did not improve their performance in the final year to the same extent as their White and Asian counterparts.

In support of our third hypothesis, the BME achievement gap was markedly smaller in students who had taken a placement year. Placement experience was also associated with a reduced gap in the final year between those with higher vs. lower entry tariffs – a finding particularly true for males. Students who took placements improved their marks more in the final year than those who did not. Previous research has shown the beneficial effects of placement experience on final year performance (e.g., Reddy and Moores, 2006, 2012; Jones et al., 2015), but the current study extends this work to suggest that placement experience is associated with reduced achievement gaps – for both BME students and for students entering University with different levels of prior achievement. Placement experience may therefore offer a mechanism to help bridge the BME achievement gap, although it does not eliminate it.

Contrary to our final hypothesis, the size of the BME achievement gap did not differ between students of higher and those of lower prior attainment. Prior attainment is therefore not likely to be able to account for the different sizes of BME attainment gaps reported across different types of institutions (Fielding et al., 2008).

In addition, after controlling for entry tariff and other variables, we did not find a significant difference in the performance of males vs. females. Previous literature has demonstrated superior attainment in female students (e.g., Broecke and Nicholls, 2007; Thiele et al., 2016). However, in contrast to the males, female achievement was higher if their parents had been to University; Mehta et al. (2011) discuss a range of reasons why first generation students find study more difficult. Also, in contrast to males, females showed an overall main effect of year of study, with an uplift in their grades in their final year. However, as already discussed, Black females who entered University with low UCAS excellence and who did not do a placement did not show this uplift. Cotton et al. (2016) suggested that male (and overseas BME) students may be more likely to overestimate their likely degree outcomes, possibly leading to an under-commitment of study time. Female students were reported to be generally more anxious about their studies and placed greater emphasis than males on the academic (vs. social) aspects of University life, although some reporting bias may have been evident. Richardson (2008) found that the BME attainment gap was more pronounced in women than men, but our data did not show this pattern overall.

A study by the National Union of Students (2011) “Race for Equality” proposed a number of possible reasons for the BME attainment difference including previous educational experience, teaching and learning factors within the institution, institutional environment and ‘broader’ (psychological) issues. Cotton et al. (2016) also considered differences in learning approaches (see also Ridley, 2007; Richardson, 2010), integration into University life (see also Eimers and Pike, 1997; Severiens and Wolff, 2008; Meeuwisse et al., 2010a; Stuart et al., 2011) and having an accurate understanding of achievement levels. Cotton et al. (2013) also found that BME (and male) students were more likely to have part time jobs during term time (see, e.g., Moreau and Leathwood, 2006 for a discussion of the risk of term time working exacerbating inequalities). Meeuwisse et al. (2010b) provide evidence that BME students who withdraw from higher education more often report doing so because of a perceived lack of quality of education rather than a lack of ability (see also Eimers and Pike, 1997). Thus, perceptions of quality may also have a greater impact on the motivation of BME students. A SOAS Students’ Union (2016) report, based on qualitative data, takes a somewhat more critical ‘non-deficit’ stance, suggesting exclusion and discrimination in the teaching and learning environment contributes to the gap. Indeed, a recent HEFCE (2016) report suggests that BME graduates are more likely to wish they had made different Higher Education choices. Richardson (2015) provides a useful summary of ‘what we know and what we don’t know’ about the under-attainment of BME students in United Kingdom higher education and suggests that ethnicity is a proxy for other factors yet to be identified, which are confounded with ethnicity.

## Limitations and Conclusion

Since students in this study were not randomly allocated to take a placement (or not), any causal inferences regarding placements reducing the BME achievement gap cannot be made. A fully randomized study would not be possible. Jones et al. (2015) discussed the self-selection issue in terms of students’ choice whether or not to take a placement year and found that, although some self-selection is present, the impact of placement experience is still positive. A number of demographic factors – including ethnicity – are known to be associated with the likelihood of a student taking an optional placement at Aston University. Of concern, therefore, is that the reduced likelihood of BME students taking placements also means that, if placement experience can act to reduce achievement gaps, the students that may benefit most from this experience are also those least likely to use the opportunity. Encouraging BME students to participate in optional placement experience may therefore be one way of helping to reduce the BME achievement gap.

The observational nature of our study does not allow us to infer what the cause of the BME achievement gap at Aston University might be. However, what has been shown is that the gap still exists even after statistically controlling for a number of demographic and situational variables. It is also present across 3 years of study. Although our findings may be somewhat less generalisable to other institutions due to the particular diverse nature of the student population at Aston University, conversely they can be taken as a strong indication that the BME achievement gap is not likely to be *fully* accounted for by the experience that BME students are often a minority in United Kingdom universities. However, this is not necessarily to say that being a minority would not present issues that might further contribute to any gap. Furthermore, although the student population at Aston University may be ethnically diverse, the staff profile is markedly less so – only 5% of Aston University’s academic staff are BME. The lack of BME staff would limit exposure to role models of the same ethnicity which may have some impact on motivation and success (e.g., Connor et al., 2004).

In this study, we only investigated the influence of three broad categories of ethnicity – White, Black and Asian – on performance. Thus, as well as omitting students from the other broad categories, we also ignored potentially significant and important differences within those broad categories. Although this strategy allowed us to ensure a relatively large sample size in each category, it will undoubtedly have also meant that important differences were ignored. Nevertheless, even considering these three broad categories, we observed different patterns of performance and different influences on performance, suggesting that the BME achievement gap is likely to be modulated by a number of factors acting differently on different groups. There were variables which would have been useful to include in the model, but for which no data were available, including term-time working, parental income and English as an Additional Language. Students with BTEC qualifications, and students from independent schools were excluded from analyses, yet these students could have contributed to the gap, despite the low numbers in each group. A further limitation was that we deliberately included socioeconomic status and UCAS entry tariff as covariates in the model in order to provide a statistical control for these factors, but ethnicity is not statistically independent from socioeconomic status or entry tariff. Indeed, in our sample, Asian students were more likely than Black students to be categorized as high UCAS excellence and there was a significant association between socioeconomic status and ethnicity.

The sizes of the effects reported in the study appear relatively small. In terms of final year average grades, the effect of ethnicity accounted for just 1.4% of the total variance. However, to provide some context, the effect of prior attainment was just 1.8% of the total variance. The mean difference between White, Asian and Black average marks was between 2 and 3 marks. Although this may not seem large, a whole degree classification spans only 10 marks and with mean values of BME groups falling toward the lower end of the 2.1 degree classification range, this magnitude of difference will very easily create differences in final degree outcomes for large numbers of students. These findings therefore have serious and long term implications. Many graduate-level jobs and post-graduate courses (and related bursaries) have a 2.1 classification degree or above as a minimum entry requirement. This means that BME graduates are less likely to be able to benefit from these opportunities, impacting on both their career and further educational opportunities (e.g., Naylor et al., 2007). Although Aston University has an excellent reputation in achieving good graduate outcomes for its students, universities also have a moral and civic responsibility to provide equality of opportunity and outcomes for students from all backgrounds.

Our findings therefore reinforce the existence of the BME achievement gap in final year performance in a United Kingdom University and – for the first time – show that a gap exists even in a University with a predominantly non-white student demographic. We also show that the achievement gap is also present from the first year of study and remains reasonably constant for most groups. However, placement experience is associated with a smaller – but still present – BME achievement gap in the final year. Future research should attempt to evaluate the impact of work placements on the BME achievement gap in other institutions and try to further disentangle potential self-selection effects of participating in a placement from the benefits offered from the placement itself. However, placements are important for both career and degree outcomes, particularly for students with certain demographic characteristics and prior performance profiles and BME students should be encouraged to gain such experience. Higher Education Institutions need to invest in resources to motivate hard- to-reach groups and in particular students who enter university with weaker prior achievement. Although Aston University will doing exactly that, completely eliminating the BME achievement gap will clearly involve going beyond anything which we already do.

## Ethics Statement

The data for this study were part of a larger dataset collected by the University for various functions including equal opportunities monitoring and enhancing the student experience. No new or additional data were collected and all data were anonymized. The analyses conducted were part of a project which monitors fair access and success. Students consent to their data being used for these purposes upon accepting their offer to study.

## Author Contributions

All authors listed have made a substantial, direct and intellectual contribution to the work, and approved it for publication.

## Conflict of Interest Statement

The authors declare that the research was conducted in the absence of any commercial or financial relationships that could be construed as a potential conflict of interest.

## Footnotes

1 The Russell Group is a United Kingdom group which claims to represent 24 leading research intensive Universities. The Universities in the group are generally considered highly prestigious and highly selective in their intake.

2 A group of Universities which describes itself as “The Association for Modern Universities in the United Kingdom.”
